# Vegetation Delight? Greenness and Reduced Risk of Nonaccidental Death

**DOI:** 10.1289/ehp.124-A169

**Published:** 2016-09-01

**Authors:** Carrie Arnold

**Affiliations:** Carrie Arnold is a freelance science writer living in Virginia. Her work has appeared in *Scientific American*, *Discover*, *New Scientist*, *Smithsonian*, and more.

Although more than half of humanity now lives in urban areas,^1^ we have by and large retained a love for green spaces. A growing body of research suggests that exposure to “greenness” (i.e., vegetation) can improve both physical and mental health.^2^ However, studies linking the greenness of an area to mortality have been limited in looking at populations over time. Environmental epidemiologist Peter James and his colleagues at the Harvard T.H. Chan School of Public Health addressed this gap with a long-term study of U.S. women. They found that living in more densely vegetated areas was associated with fewer deaths from causes other than accidents.^3^

The researchers began with data from the Nurses’ Health Study. This prospective study began in 1976 and enrolled 121,701 registered nurses aged 30–55. Scientists have tracked more than 90% of participants since then, and by the time of the current study, at least 10 nurses lived in each of the 48 contiguous states.

**Figure:**
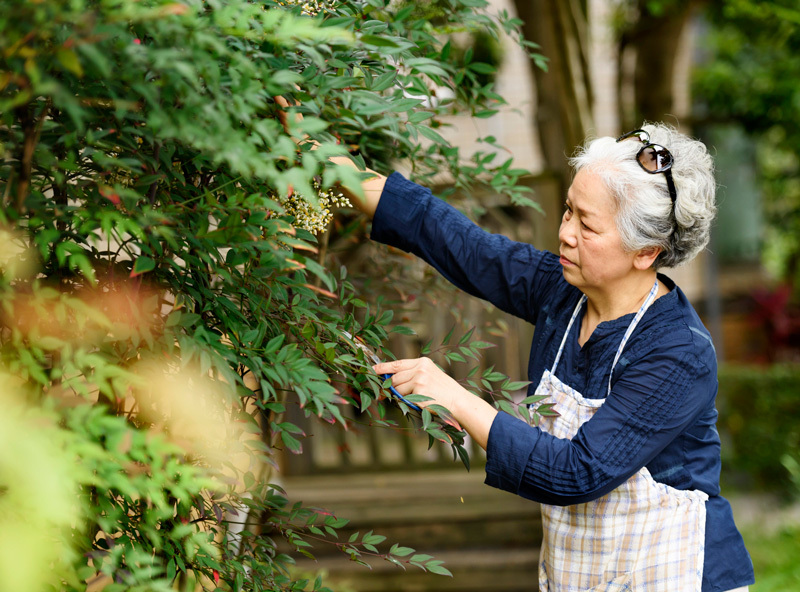
A long-term study of women across the contiguous United States found that women whose homes were surrounded by more dense vegetation were less likely to die from a variety of diseases. © Leren Lu/Getty Images

James and colleagues characterized the vegetation near each participant’s address using the Normalized Difference Vegetation Index. This index calculates the presence and density of living green plants in a given area as a function of the wavelength of light reflected by the area into outer space. The researchers considered measures of women’s cumulative exposure to greenness over time and their short-term exposure based on greenness at specific times during the study.

After controlling for factors including socioeconomic status, race, smoking, and whether the women lived in a rural or urban area, the researchers estimated a 12% lower rate of nonaccidental death between women who lived in the most densely versus least densely vegetated areas. When looking at specific causes of death, the researchers estimated a 41% lower rate of kidney disease mortality, a 34% lower rate of respiratory disease mortality, and a 13% lower rate of cancer mortality in the women who lived in the greenest areas, compared with those in the least green areas.^3^

“We were pretty surprised at the magnitude of the association between increased greenness exposure and the decrease in mortality,” James says.

The study of greenness as a predictor of health is still very young, but James says researchers are beginning to work out the various pathways by which exposure to vegetation may improve health. The availability of attractive green spaces may spur people to spend more time outdoors, which is associated with reduced risk of obesity^4^ and cardiovascular disease.^5^ Other studies have found that trees may reduce levels of air pollutants that contribute to cardiovascular and respiratory diseases.^6^ Lastly, research from the field of ecopsychology shows that being around vegetation can provide substantial mental health benefits that may, in turn, lead to better physical health.^7^ ^,^ ^8^ According to the authors’ own analysis, vegetation appeared to act in part through beneficial effects on mental health and social engagement, and possibly by reducing air pollution and promoting physical activity.^3^

One limitation of this study is that the participants in the study were all female and largely white. “While generalizing the results of this study to the entire U.S. population is difficult, as this research includes only nurses, this study helps open up the ‘green box’ of green space benefits,” says Perry Hystad, an environmental epidemiologist at Oregon State University. Hystad was not involved in the study.

There is evidence that exposure to greenness may provide even more health benefits to children, the elderly, and less affluent people.^9^ ^,^ ^10^ ^,^ ^11^ However, disadvantaged people in urban areas tend to have less access to safe green spaces.^12^ ^,^ ^13^ “Green space in communities may be a big environmental justice issue,” Hystad says.

The results of the new study are consistent with other findings and add to the body of evidence that greenness promotes health, says University of Washington epidemiologist Howard Frumkin, who was not involved in the study. “Exposure to green space prevents or ameliorates a number of diseases, it’s relatively free of side effects, it’s inexpensive, and it doesn’t need a medical professional to supply it,” Frumkin says. “So this is a truly exciting public health strategy.”
